# The Effects of Sensorineural Hearing Impairment on Asynchronous Glimpsing of Speech

**DOI:** 10.1371/journal.pone.0154920

**Published:** 2016-05-04

**Authors:** Erol J. Ozmeral, Emily Buss, Joseph W. Hall

**Affiliations:** 1 Department of Communication Sciences and Disorders, University of South Florida, Tampa, Florida, United States of America; 2 Department of Otolaryngology/Head and Neck Surgery, School of Medicine, University of North Carolina, Chapel Hill, North Carolina, United States of America; University of Modena and Reggio Emilia, ITALY

## Abstract

In a previous study with normal-hearing listeners, we evaluated consonant identification masked by two or more spectrally contiguous bands of noise, with asynchronous square-wave modulation applied to neighboring bands. Speech recognition thresholds were 5.1–8.5 dB better when neighboring bands were presented to different ears (dichotic) than when all bands were presented to one ear (monaural), depending on the spectral width of the frequency bands. This dichotic advantage was interpreted as reflecting masking release from peripheral spread of masking from neighboring frequency bands. The present study evaluated this effect in listeners with sensorineural hearing loss, a population more susceptible to spread of masking. Speech perception (vowel-consonant-vowel stimuli, as in /*a*B*a*/) was measured in the presence of fluctuating noise that was either modulated synchronously across frequency or asynchronously. Hearing-impaired listeners (n = 9) and normal-hearing controls were tested at either the same intensity (n = 7) or same sensation level (n = 8). Hearing-impaired listeners had mild-to-moderate hearing loss and symmetrical, flat audiometric thresholds. While all groups of listeners performed better in the dichotic than monaural condition, this effect was smaller for the hearing-impaired (3.5 dB) and equivalent-sensation-level controls (3.3 dB) than controls tested at the same intensity (11.0 dB). The present study is consistent with the idea that dichotic presentation can improve speech-in-noise listening for hearing-impaired listeners, and may be enhanced when combined with amplification.

## Introduction

Recognizing speech in a spectro-temporally dynamic background relies, in part, on a listener’s ability to integrate speech cues from the time/frequency regions where the signal-to-noise ratio (SNR) is favorable [1–6]. The ability to use isolated segments of the speech stream, sometimes referred to as speech “glimpsing” [4], is enhanced in the presence of masker fluctuation relative to steady maskers. The benefit associated with this masker fluctuation has been called the fluctuating masker benefit [7, 8] or masking release [9]. Hearing-impaired (HI) listeners are often shown to have less ability to benefit from dynamic changes in local SNR compared to normal-hearing (NH) listeners. Though the mechanisms responsible for this are not fully understood, a number of factors associated with hearing loss have been implicated, including reductions or deficiencies in: audibility [10], temporal resolution [10–12], frequency selectivity [13–17], temporal fine structure processing [18–20], across-frequency integration [21, 22], and effects related to SNR in the baseline condition [7]. The purpose of the present study was to specifically asses the roles that frequency selectivity and audibility have on HI listeners’ limited ability to benefit from masker fluctuation for speech perception.

We used a unique masking release paradigm with temporally-modulated noise maskers that were either comodulated or uncomodulated across frequency [2]. So as not to be confused with a well-established psychoacoustic phenomenon, comodulated masking release (CMR [23]), we refer to the comodulated and uncomodulated maskers as synchronously- and asynchronously-modulated maskers, respectively. One motivation for testing these unique maskers is that asynchronously-modulated maskers are more ecologically relevant than synchronously-modulated maskers, as many natural listening environments contain multiple sound sources. However, the periodic modulation pattern and constant level of the asynchronously-modulated maskers does not capture the variability and unpredictability associated with many real-world sounds. Masking release associated with these modulated maskers has previously been reported for NH listeners [2, 24], but not for HI listeners, who are likely to show less masking release than NH listeners [7, 10–22].

Our previous work showed that glimpsing in spectro-temporally complex environments improves when negative effects of masking spread are removed [24]. For asynchronously-modulated maskers, in particular, potentially favorable SNRs in spectro-temporal regions of masker minima are influenced by neighboring spectro-temporal regions of masker maxima. The severity of masking spread is dependent on the frequency selectivity of the individual. This was previously assessed by measuring performance in the asynchronously-modulated masker presented either monaurally or dichotically. Dichotic presentation meant that alternating frequency regions were separated across the ears to avoid peripheral masking spread from proximal frequency regions [25–29]. Masking release in dichotic, asynchronously-modulated masker conditions was larger than in the monaural case, and this was interpreted as the direct result of removing negative effects of masking spread. Recent work by Stone and colleagues [8], however, suggests that dichotic presentation could have reduced masking by eliminating intermodulations resulting from an interaction between masker bands in the periphery. Nevertheless, the potential for a benefit in HI listeners from dichotic presentation has not been evaluated for these maskers, and results may provide further support for the role of frequency selectivity in masked speech perception.

A number of studies have indicated that frequency selectivity is often reduced in listeners with sensorineural hearing loss [30–33]. One manifestation of reduced frequency selectivity in hearing impairment is a greater effect of spread of masking [31, 34, 35], although not all studies have observed consistent differences between listeners with and without hearing loss ([36], for a review, see [37]). The HI listeners in the present study were expected to experience a robust benefit from dichotic presentation of stimuli in the asynchronously-modulated masker condition. Such a result would be consistent with an interpretation that poor frequency selectivity limits glimpsing in spectro-temporally complex backgrounds. We also considered the possibility that HI listeners would have a limited ability to integrate information across frequency. Some support for this possibility was reported by Healy and Bacon [21] and Healy and Carson [22], although this deficit has not been seen in all paradigms [5]. If such limitations are present for HI listeners, it is unclear whether these factors would limit the extent to which dichotic presentation could help performance via reduced spread of masking. We included control conditions to evaluate spectro-temporal integration, allowing us to test whether HI listeners have comparable integration abilities to those seen in NH listeners [24]. Finally, the role of audibility was separately evaluated by including normal-hearing control listeners who received either equal intensity or equivalent sensation level as the HI test group.

The goal of the present study was to assess HI listeners’ speech perception in conditions where masking spread could be alleviated through a dichotic manipulation. Results showed that HI listeners were susceptible to limits in masking release overall, believed to be an effect of poor audibility. Nonetheless, dichotic listening was demonstrated to be beneficial for HI listeners under some masking conditions, thereby leaving the possibility that frequency selectivity was a viable target for remediation. In addition, these results offer further insight into HI listeners’ abilities to integrate speech glimpses across time and frequency [5, 22], and under certain scenarios, we believe dichotic presentation may lead to better speech perception for bilateral hearing-instrument users.

## Materials and Methods

### Ethics Statement

All testing followed the ethical guidelines provided by the National Institutes of Health of the United States of America. Subjects provided written informed consent prior to all test measures and were compensated for their participation. The study, including consent and compensation, was approved by the Institutional Review Board at the University of North Carolina at Chapel Hill.

### Listeners

**Fig 1:**
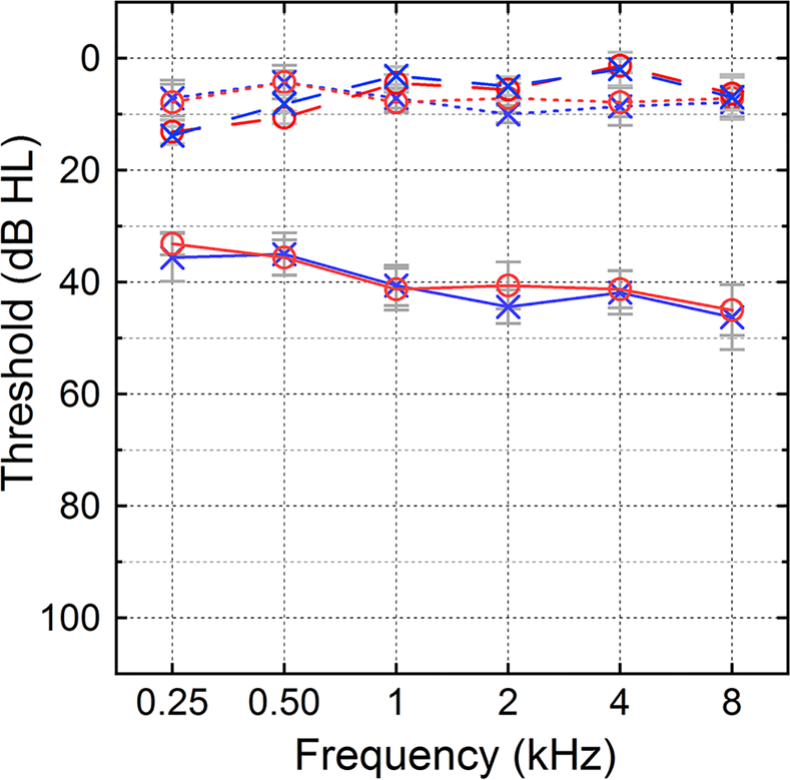
Pure-tone audiometry for participants. Audiograms for normal-hearing (NH; n = 7; dotted lines), normal-hearing at equivalent sensation level (NH_SL_; n = 8; dashed line), and hearing-impaired (HI; n = 9; solid lines) listeners for both left (blue X’s) and right (red circles) ears in dB HL. The HI listeners were screened to have roughly flat and symmetric mild-to-moderate hearing losses. Error bars represent one standard error of the mean.

Twenty-four native English-speaking adults were recruited from the local and surrounding communities. The HI group (n = 9) received the same stimuli as the normal-hearing control group (NH, n = 7), and additional data were collected from a secondary, normal-hearing group (NH_SL_, n = 8) who received stimuli near the sensation level of the HI group. The NH and NH_SL_ listeners had pure-tone thresholds of 20 dB HL or lower at octave frequencies from 0.25 to 8 kHz in each ear [38]. The HI listeners had bilateral mild-to-moderate sensorineural hearing loss of no more than 60 dB HL between 0.25 and 8 kHz. Thresholds were approximately symmetric (≤ 20 dB difference between ears) and relatively flat (≤ 25 dB difference between 500 and 4000 Hz in all but one ear). Flat hearing loss was desirable to ensure approximately comparable access to speech cues across the speech spectrum. Ages ranged from 21 to 68 years old and were roughly matched across NH and HI groups (NH group: mean 42.9 yrs ± 14.4 sd; HI group: mean 46.6 yrs ±17.4 sd). Because the NH_SL_ group was a secondary dataset, no attempt was made to match age with the two primary groups (mean 29.5 yrs ± 13.5 sd). Listeners over the age of 60 years (1 NH and 2 HI listeners) completed a cognitive assessment before the experiment (Montreal Cognitive Assessment; [39]). These older listeners were required to obtain a score of 26 or better for inclusion in the study, and all three met this criterion. Demographic information is reported in the left-most columns of Table 1, and Fig 1 presents the average audiograms for each group.

**Table 1:** Individual age and audiometric data.

**Group ID**	**Age (yrs)**	**PTA-L (dB)**	**PTA-R (dB)**	**VCV in Quiet (dB)**
HI02	34.8	43.3	50.0	n/a
HI08	21.2	55.0	55.0	n/a
HI09	56.1	43.3	40.0	59.6
HI10	53.7	41.7	38.3	57.5
HI13	41.5	45.0	41.7	67.0
HI14	23.6	35.0	30.0	51.3
HI16	68.9	33.3	28.3	48.4
HI17	67.1	23.3	30.0	46.2
HI18	52.8	40.0	45.0	63.8
MEAN (sem)	46.6 (5.8)	40.0 (2.9)	39.8 (3.1)	56.2 (3.0)
**Group ID**	**Age (yrs)**	**PTA-L (dB)**	**PTA-R (dB)**	**VCV in Quiet (dB)**
NH01	18.4	13.3	15.0	28.0
NH03	44.8	6.7	6.7	33.6
NH04	38.1	3.3	-3.3	30.4
NH06	48.3	8.3	3.3	28.9
NH07	45.0	8.3	6.7	26.7
NH11	38.8	1.7	5.0	28.5
NH12	66.8	8.3	11.7	37.8
MEAN (sem)	42.9 (5.4)	7.1 (1.4)	6.4 (2.2)	30.6 (1.5)
**Group ID**	**Age (yrs)**	**PTA-L (dB)**	**PTA-R (dB)**	**VCV in Quiet (dB)**
NH_SL_19	47.5	5.0	1.7	n/a
NH_SL_20	54.3	10.0	5.0	n/a
NH_SL_21	20.5	3.3	8.3	23.4
NH_SL_22	25.6	5.0	8.3	25.0
NH_SL_23	24.6	6.7	13.3	28.7
NH_SL_24	21.5	6.7	5.0	27.9
NH_SL_25	21.3	1.7	5.0	24.6
NH_SL_26	20.5	5.0	8.3	31.1
MEAN (sem)	29.5 (4.8)	5.4 (0.9)	6.9 (1.2)	26.8 (1.2)

PTA-L, pure-tone average in left ear; PTA-R, pure-tone average in right ear; VCV, vowel-consonant-vowel threshold; HI, hearing-impaired; NH, normal-hearing; NH_SL_, normal-hearing with equivalent sensation level; sem, standard error of the mean

### Stimuli

Speech stimuli were identical to those used in an earlier study [24]. The speech material included five recordings each for 12 vowel-consonant-vowels ([b d f g k m n p s t v z] as in /*aga*/), spoken by an adult female speaker and recorded at a sampling rate of 44.1 kHz. Stimulus duration ranged from 528 to 664 ms, with a mean duration of 608 ms. Each token was normalized to equal root-mean-square level and filtered into 2, 4, 8, or 16 frequency bands using sixth-order Butterworth band-pass filters. For a given number of bands, filter bandwidths were equivalent in logarithmic units, with bands spanning 0.1 to 10 kHz.

**Fig 2:**
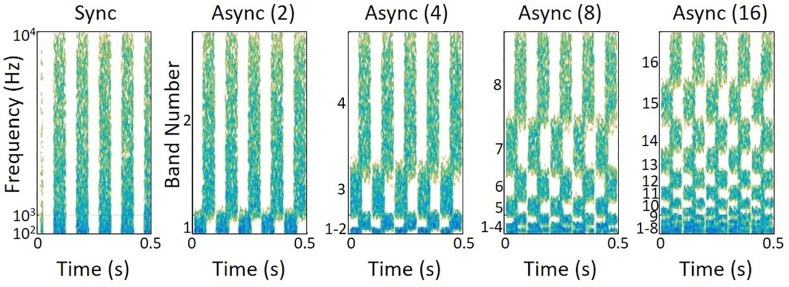
Spectrograms of modulated maskers used in the glimpsing task. From left to right, the synchronously-modulated masker (Sync) and the asynchronously-modulated masker (Async) with 2, 4, 8, or 16 numbers of frequency bands. Modulation rates were set at 10 Hz, and initial phase of modulation was random.

As in previous studies using these methods, maskers were based on broadband pink noise samples which, by definition, contained equal energy per octave band. Each masker sample was generated digitally with duration equal to the longest possible speech token plus 300 ms (964 ms total duration). Speech stimuli began 150 ms after the onset of the noise masker. Masker modulation was either synchronous (Sync) or asynchronous (Async). Spectral representations of the modulated maskers are depicted in Fig 2, including asynchronously-modulated maskers with increasing numbers of filtered frequency bands. Sync maskers were modulated in the time-domain with a 10-Hz quasi-square wave with a random starting phase; 10-ms raised cosines were used to smooth level transitions and limit spectral splatter. To create Async maskers, the pink noise was filtered into 2, 4, 8, or 16 bands using sixth-order Butterworth band-pass filters. Then a 10-Hz quasi-square wave was applied to each noise band via multiplication. A single, randomly selected starting phase was chosen for the odd-numbered bands, and the inverse phase was used for the even-numbered bands. Bands were numbered by frequency region, beginning with the lowest frequency band. Before stimulus presentation, speech and noise signals were up-sampled to 48828 Hz to conform to hardware specifications (Tucker-Davis Technologies, Alachua, FL).

Either monaural (left [L] or right [R] ear only) or dichotic (D) stimuli were presented in a single block of trials. Monaural stimuli consisted of combined speech and noise signals. Dichotic stimuli included the odd-numbered bands of the combined speech and noise presented to the left ear, and even-numbered bands of the combined speech and noise presented to the right ear. In some cases, masker bands were presented to a single ear without the associated speech bands (see dichotic control conditions described below).

### Procedure and conditions

Procedures of the speech identification task were similar to those used in a previous study [24]. On each trial, speech tokens were randomly selected with replacement, and the task was to indicate the consonant that was heard by selecting one of the 12 alternatives on the computer screen using a mouse. The speech recognition thresholds (SRTs) were measured using an adaptive up-down tracking algorithm using 4 dB steps, which estimated 50% correct identification [40]. The SRTs were determined based on the last 24 of 26 track reversals. This procedure was controlled using a custom Matlab (Mathworks, Inc., Natick, MA) script. Stimuli were presented through a pair of insert earphones (Etymotic ER-2, Elk Grove Village, IL), and listeners were seated in a single-wall, sound-treated booth. The first block of trials was a speech identification task with no masking. This served both to familiarize listeners with the task, and as a measure of in-quiet SRTs (results reported in Table 1). For the first two hearing-impaired and two normal-hearing listeners, in-quiet SRTs are not available due to an initial requirement of 100% speech identification accuracy at a comfortably loud presentation level. This initial requirement was subsequently replaced with an in-quiet threshold measure of speech reception.

For testing in quiet, thresholds were obtained by adjusting the level of the signal. Subsequent blocks of trials contained a masker. In these trials the SNR was adjusted adaptively, starting with an initial SNR of 10 dB. In the unmodulated noise condition, the level of the stimulus (signal plus masker) was fixed at 85 dB SPL for the HI and NH groups, and it was attenuated to 55 dB SPL for the NH_SL_ group. To achieve the fixed level with varying SNR, two scalars were generated–one for the target, to produce the desired SNR, and the other for the summed target-plus-masker, to control the overall level. Both scalars were generated based on a bandpass filtered target (0.1–10 kHz) and a steady noise masker sample. This same procedure, generating a total level of 85 dB SPL, was the first stage for all other masked speech identification conditions. For the synchronous modulation, the noise was bandpass filtered and subsequently amplitude modulated. For the asynchronous monaural conditions, the noise was filtered and modulated before presentation. For the asynchronous dichotic conditions, the noise was modulated on a band-by-band basis prior to presentation. The full intensity was consequently reduced 3.2 dB by amplitude modulation in the Sync and Async conditions, and reduced further, in an ear-specific way, in the dichotic conditions. Trials were blocked by condition, and the order of conditions was quasi-randomly selected for each listener to avoid order effects. Each listener performed either three or four tracks for each condition. The fourth estimate was obtained if the first three thresholds were not all within 3 dB of each other. Overall testing time was roughly 5 h, typically spread out over five sessions on multiple days.

**Fig 3:**
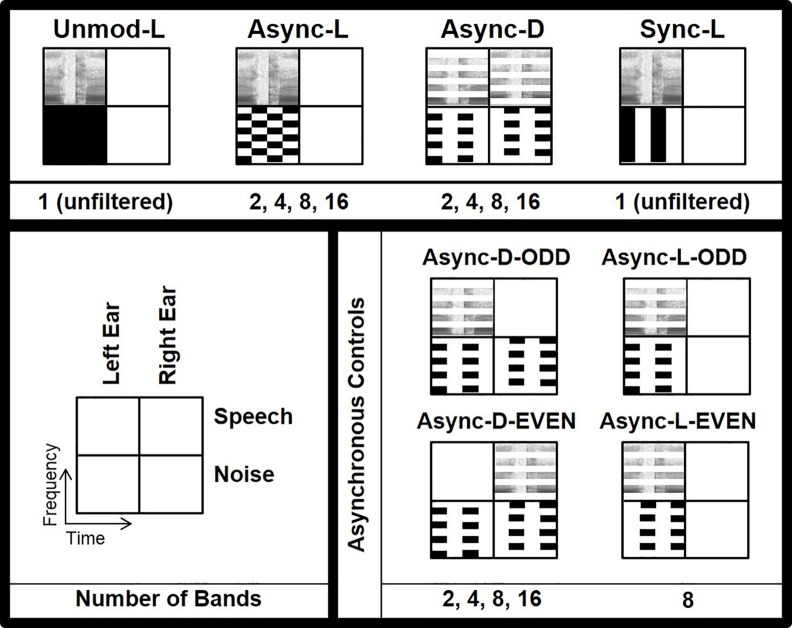
Schematics of monaural and dichotic masking conditions. Primary conditions are represented on the top row, and control conditions are shown below. Only left-ear (L) schematics are visually depicted for monaural conditions, but right (R) ear conditions were also tested. As the legend indicates, each condition is represented as a 2-by-2 box in which the left and right columns represent stimulation of the left and right ears, respectively, and the top and bottom rows represent the speech and noise stimuli, respectively. In each box, frequency from 0.1 to 10 kHz is represented vertically, and a time span of 200 ms is represented horizontally. Speech is represented via spectrogram, and noise is represented in black. Amplitude modulation is performed at a rate of 10 Hz, and frequency bands are equally spaced on a logarithmic scale. The order of the primary conditions in the top row is an indication of the expected ranking in thresholds, with the worst performance starting on the left, with the Unmod-L and Unmod-R conditions, and the best performance on the right, with the Sync-L and Sync-R conditions. The numbers of bands tested per condition are given below each condition schematic.

Fig 3 illustrates the key features of the 28 total conditions described in the remainder of this paragraph. In the baseline conditions, unmodulated noise was presented monaurally to either the left or right ear (Unmod-L and Unmod-R). The Sync condition was presented monaurally to each ear as well (Sync-L and Sync-R). For each Async monaural and dichotic condition (Async-L, Async-R and Async-D, respectively), stimuli were processed into 2, 4, 8, or 16 bands for a total of twelve Async test conditions. Additionally, there were two control conditions for the Async-D conditions. The first set of control conditions presented the Async-D masker (with 2, 4, 8, or 16 bands) but included only half of the speech bands: in Async-D-EVEN, the even-numbered speech bands were presented to the right ear, and in Async-D-ODD, the odd-numbered speech bands were presented to the left ear. These control conditions were intended to reveal whether performance in the Async-D conditions could be accounted for solely by either the even or odd speech bands alone. By including the masker in both ears but speech in only one ear, we were also able to test the possibility that contralateral maskers could affect performance. Two additional control conditions were included to assess masking in the Async-D condition from a single ear. In the Async-L-ODD and Async-L-EVEN conditions, only the odd-numbered or even-numbered frequency bands were presented to the left ear, respectively (the right-ear conditions were also tested but are not depicted in Fig 3). These conditions were only run using 8 band-pass filters (i.e., 4 bands per ear). All conditions were tested for the HI and NH groups, whereas the NH_SL_ group was tested only on conditions with 4 or 8 bands.

### Data analysis

Although we tested monaural conditions in both left and right ears, the performance obtained from each of the two ears was very closely matched in both the NH or HI groups. For example, the absolute value of the difference in SRTs for the Unmod-L and Unmod-R conditions was (on average) 0.8 dB for NH listeners and 1.2 dB for HI listeners. Given the similarity across ears, data were analyzed after taking the average of the left and right ear SRTs. The resulting values are identified with an ‘M’ (e.g., Unmod-M) to indicate monaural presentation. Moreover, reporting of data in the control conditions was limited to the best-case performance. For instance, on a subject-by-subject basis, the better threshold in either the Async-D-ODD or Async-D-EVEN was the only dichotic control threshold used to assess the performance on control conditions. The better of the two dichotic control conditions is reported as Control-D, and the better of the monaural control conditions is reported as Control-M. We used the lower (better) of the two control thresholds to evaluate performance in the primary Async conditions because it would provide the most conservative measure of integration when all bands were available. Additionally, this simplification mitigates effects related to subtle asymmetries in hearing between ears.

Data in each test condition were submitted to a Shapiro-Wilk test of normality. Significant values were obtained in only two cases: the 16-band, monaural Async condition for the NH group (p = 0.001) and the 8-band, dichotic Async condition for the NH_SL_ group (p = 0.01). It was decided to conduct parametric analyses despite evidence of non-normality in these two conditions due to simplicity and ease of interpretation.

## Results

### Hearing-impaired listener thresholds

**Fig 4:**
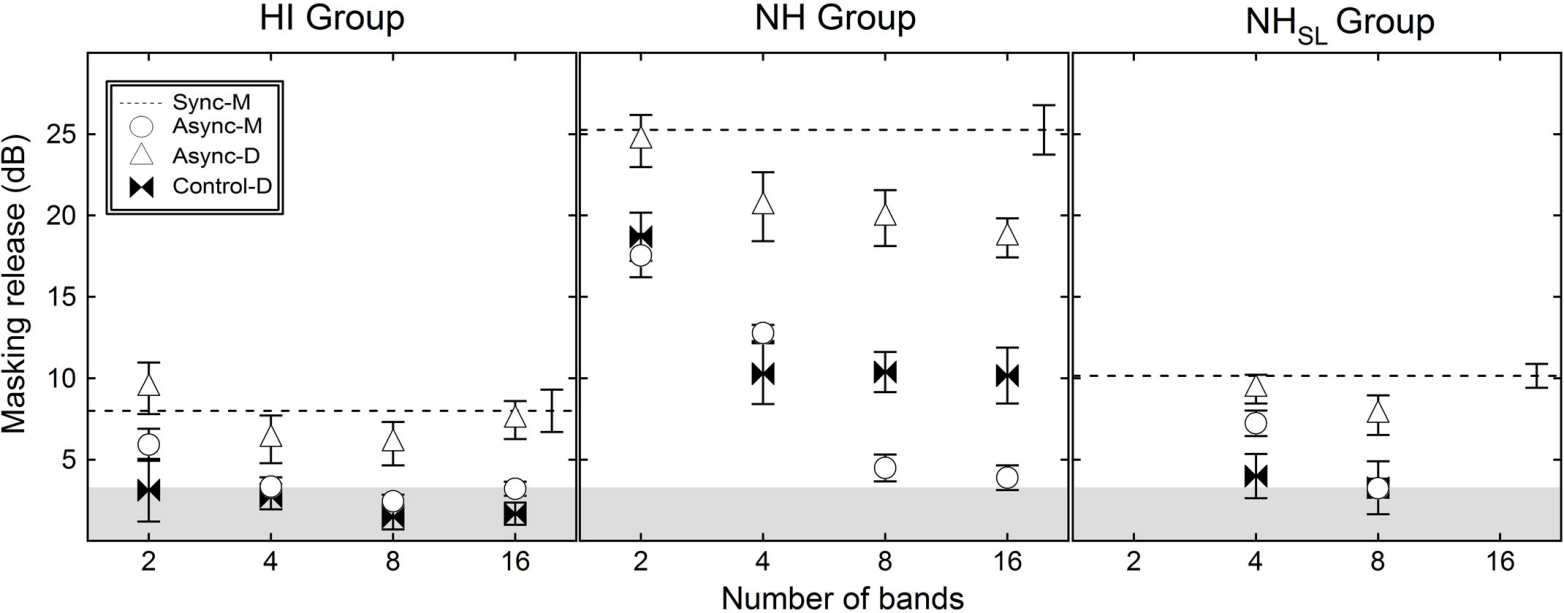
Masking release relative to unmodulated noise. Mean masking release is plotted for modulated noise conditions relative to the unmodulated condition for hearing-impaired (HI; left panel) normal controls with stimulus presentation at either the same intensity (NH; middle panel) or same sensation level (NH_SL_; right panel). The difference in mean thresholds relative to the Unmod-M condition at 2, 4, 8, and 16 bands are plotted for the monaural asynchronous condition (Async-M; circles), the dichotic asynchronous condition (Async-D; triangles), the better of the dichotic control conditions (Control-D; bowties), and the mean of the synchronous conditions (Sync-M; dashed line). Error bars indicate standard error of the mean (n = 9 for HI group; n = 7 for NH group; n = 8 for NH_SL_ group). The shaded region at the bottom of each panel indicates the range over which masking release might be accounted for by reductions in masker level associated with amplitude modulation (3.2 dB).

Mean SRTs for the HI listeners are presented in Table 2 (top) for all primary conditions and the better of the control conditions. To measure the ability to glimpse speech in a fluctuating masker, data were analyzed in terms of masking release, quantified as the difference in SRT between a condition with modulated noise and the Unmod-M condition. Fig 4 (left panel) shows masking release (in dB) for the average of the monaural Async conditions (Async-M), the dichotic condition (Async-D), the average of the Sync conditions (Sync-M), and better of the Async-D control conditions (Control-D), expressed relative to the SRT for the Unmod-M reference value. Error bars show one standard error of the mean, and symbols indicate the masker condition, as defined in the legend. The shaded region at the bottom of the figure indicates the range of values that can be accounted for by the fact that modulation reduces the overall masker level by 3.2 dB. The masking release for HI listeners was greatest for Sync-M (average of 8.0 dB) and for Async-D (ranging from 6.0 to 9.4 dB), but it was consistently smaller for Async-M (ranging from 2.4 to 5.9 dB). Masking release was evaluated with single-sample one-tailed t-tests, with a reference of 3.2 dB (the reduction in masker level associated with modulation). Masking release was greater than 3.2 dB for all four Async-D conditions and for the 2-band Async-M condition (p < 0.05), but not for the other Async-M conditions (p ≥ 0.421). A two-way repeated-measures ANOVA was performed to compare performance in the Async-D and Async-M conditions, with two levels of condition and four levels of band number. This analysis yielded a main effect of condition (F_1,8_ = 10.2, p = 0.013), a main effect of the number of bands (F_3, 24_ = 15.7, p < 0.001), but no interaction (F_3, 24_ = 0.43, p = 0.73). Simple main effects testing was performed to compare masking release in the Async-D to the Async-M conditions; in all cases more masking release was observed in the dichotic than the monaural presentation condition (p < 0.05, with Bonferroni correction), as was the case for NH listeners in the previous study [24].

**Table 2:** Mean speech recognition thresholds (in dB SNR) for each primary test condition and the better of the control conditions.

**HI**		**Number of Bands**			
		**2**	**4**	**8**	**16**
Unmod-M	0.3 (0.5)				
Sync-M	-7.7 (1.2)				
Async-M		-5.6 (0.9)	-3.0 (0.5)	-2.1 (0.5)	-2.9 (0.4)
Async-D		-9.1 (1.3)	-6.0 (1.4)	-5.7 (1.2)	-7.1 (1.0)
Control-D		-2.8 (1.8)	-2.4 (0.7)	-1.5 (0.6)	-1.4 (0.8)
Control-M				-5.4 (1.4)	
**NH**		**Number of Bands**			
		**2**	**4**	**8**	**16**
Unmod-M	-0.3 (0.6)				
Sync-M	-25.5 (1.5)				
Async-M		-17.8 (1.3)	-13.0 (0.6)	-4.7 (1.2)	-4.1 (0.8)
Async-D		-24.9 (1.7)	-20.8 (1.8)	-20.1 (1.7)	-18.9 (1.0)
Control-D		-19.0 (1.4)	-10.6 (1.8)	-10.7 (1.1)	-10.4 (1.7)
Control-M				-14.7 (1.5)	
**NH**_**SL**_		**Number of Bands**			
		**2**	**4**	**8**	**16**
Unmod-M	-2.2 (0.6)				
Sync-M	-12.4 (1.1)				
Async-M			-9.6 (1.1)	-5.3 (1.1)	
Async-D			-11.6 (1.3)	-10.0 (1.6)	
Control-D			-4.0 (1.4)	-3.3 (1.6)	

HI, hearing-impaired; NH, normal-hearing; NH_SL_, normal-hearing with equivalent sensation level; Unmod-M, monaural unmodulated masker; Sync-M, monaural synchronously-modulated masker; Async-M, monaural asynchronously-modulated masker; Async-D, dichotic asynchronously-modulated masker; Control-M, monaural controls; Control-D, dichotic controls

### Normal-hearing listeners–Equal Intensity

Mean SRTs for NH listeners tested at 85 dB SPL are presented in Table 2 (middle) for all primary test conditions and the better dichotic control conditions. Fig 4 (middle panel) shows the mean masking release (in dB) of the NH group for the Async-M, Async-D, Sync-M, and Control-D, measured relative to the SRT for the Unmod-M reference. Error bars show one standard error of the mean, and symbols indicate the masker condition, as defined in the legend. The masking release for NH listeners was greatest for Sync-M (average of 25.3 dB), intermediate for Async-D (ranging from 18.6 to 24.6 dB), and least for Async-M (ranging from 3.9 to 17.5 dB), with some values in this last condition being consistent with a reduction in overall masker level (in contrast to glimpsing). Masking release was significantly greater than 3.2 dB for all modulated masker conditions (p < 0.001) in all Async-M and Async-D conditions *except* the Async-M-8 condition (p = 0.090) and the Async-M-16 condition (p = 0.211). A two-way repeated-measures ANOVA was performed to compare performance in Async-D and Async-M, with two levels of condition and four levels of band number. This analysis yielded a main effect of condition (F_1,6_ = 108.8, p < 0.001), a main effect of the number of bands (F_3,18_ = 64.5, p < 0.001), and an interaction (F_3,18_ = 15.1, p < 0.001). The interaction is explained by the greater separation between masking release observed in the different conditions as the band number increased. Simple main effects testing was performed to compare masking release in the Async-D to the Async-M conditions; in all cases better performance was observed in the dichotic than the monaural presentation condition (p ≤ 0.005, with Bonferroni correction).

### Normal-hearing listeners–Equal sensation level

Mean SRTs for NH_SL_ listeners tested at 55 dB SPL are presented in Table 2 (bottom). Fig 4 (right panel) shows the mean masking release (in dB) of the NH_SL_ group for the Async-M, Async-D, Sync-M, and Control-D, measured relative to the SRT for the Unmod-M reference. Error bars show one standard error of the mean, and symbols indicate the masker condition, as defined in the legend. The masking release for NH_SL_ listeners was greatest for Sync-M (average of 10.2 dB), intermediate for Async-D (4 bands: 9.3 dB; 8 bands: 7.7 dB), and least for Async-M (4 bands: 7.4 dB; 8 bands: 3.1 dB). Masking release was significantly greater than 3.2 dB for all modulated masker conditions (p < 0.01) *except* the Async-M-8 condition (p = 0.85). A two-way repeated-measures ANOVA was performed to compare performance in Async-D and Async-M, with two levels of condition and two levels of band number. This analysis yielded a main effect of condition (F_1,7_ = 34.0, p = 0.001), a main effect of the number of bands (F_1,7_ = 186.8, p < 0.001), and an interaction (F_1,7_ = 7.9, p < 0.05). As before with the NH group, the interaction is explained by the greater separation between masking release observed in the different conditions as the band number increased from 4 to 8 bands. Moreover, post-hoc testing showed greater masking release in the dichotic than the monaural presentation for both 4 and 8 bands (p < 0.005, with Bonferroni correction).

### Between-group analyses

#### Comparisons at same intensity

The SRTs in the Unmod-M case were submitted to a one-way ANOVA. This analysis showed no significant difference between the NH and HI listeners (F_1,14_ = 0.53, p = 0.48), which indicated that at an overall presentation level of 85 dB SPL, hearing impairment did not reliably affect speech recognition in steady noise. It is evident from Fig 4, however, that NH listeners had greater masking release in most modulated-noise conditions compared to the HI group. A one-way ANOVA for Sync-M masking release indicated that the difference was significant (F_1,14_ = 90.55, p < 0.001). With respect to the Async noise conditions, masking release data were submitted to a three-way ANOVA with two levels of presentation type (dichotic and monaural), four levels of number of bands (2, 4, 8, and 16), and two levels of listener group (NH and HI). This analysis showed significant main effects of presentation type (F_1,14_ = 87.4, p < 0.001), number of bands (F_3,42_ = 77.9, p < 0.001), and listener group (F_1,14]_ = 83.3, p < 0.001). There were also significant interactions between condition and band number (F_3,42_ = 12.4, p < 0.001), between condition and group (F_1,14_ = 23.6, p < 0.001), and between band number and group (F_3,42_ = 27.7, p < 0.001). Lastly, the three-way interaction was significant (F_3,42_ = 8.8, p < 0.001). Because the three-way interaction was significant, the other interactions and significant main effects should be interpreted with caution. The significant three-way interaction is best explained by a large and relatively constant difference between groups for the Async-D conditions, yet in the Async-M conditions, the difference between NH and HI listeners was large for the 2 and 4 band numbers and vanishingly small by 16 bands. Because modulation caused a drop in overall intensity of the masker by 3.2 dB, the Async-M thresholds probably did not reflect glimpsing for 4, 8, and 16 bands in the HI listeners, or for 8 and 16 bands for the NH listeners. From Fig 4, we can also see that while NH listeners tended to have less masking release in both noise conditions as the number of bands increased, HI listeners showed relatively consistent and low masking release for all numbers of bands. The absence of an effect of band number in the HI data may be influenced by the compressed range of thresholds, including a relatively small peak masking release for HI listeners in the Sync-M condition. This final point can be addressed by comparing data for HI and NH listeners at similar sensation levels.

#### Comparisons at equivalent sensation level

For the NH_SL_ group, average threshold in the baseline, Unmod-M condition was -2.2 dB SNR; that value was significantly lower than Unmod-M threshold for the HI group (F_1,15_ = 11.7, p < 0.005). The NH_SL_ and HI masking release data were submitted to a three-way ANOVA with two levels of presentation type (dichotic and monaural), two levels of number of bands (4 and 8), and two levels of listener group (NH_SL_ and HI). This analysis showed significant main effects of condition (F_1,15_ = 19.4, p = 0.001), number of bands (F_1,15_ = 35.5, p < 0.001), and listener group (F_1,15_ = 4.63, p < 0.05). There were also significant interactions between condition and band number (F_1,15_ = 5.0, p < 0.05) and between band number and group (F_1,15_ = 15.3, p = 0.001). There was no significant interaction between group and condition (p = 0.97), nor was there a significant three-way interaction (p = 0.21). The interaction between condition and band number appears to be explained by a greater rate of reduction in masking release from 4 to 8 bands in the monaural condition relative to the dichotic condition. The interaction between band number and group is apparent in the steeper decline in masking release from 4 to 8 bands for the NH_SL_ group than the HI group.

Masking release data from each condition, including the controls and Sync-M conditions, were submitted to one-way ANOVAs comparing the NH_SL_ and HI groups. The only significant difference between groups was in the 4-band Async-M condition (F_1,15_ = 16.8, p = 0.001). Inspection of each panel of Fig 4 shows that as the number of bands increases, masking release in the Async-M condition approaches floor at some point. Even when matched for sensation level, performance at floor occurs at a lower number of bands (4) in the HI group than in the NH_SL_ group, where floor performance is not reached until 8 bands.

#### Comparisons of dichotic advantage

**Fig 5:**
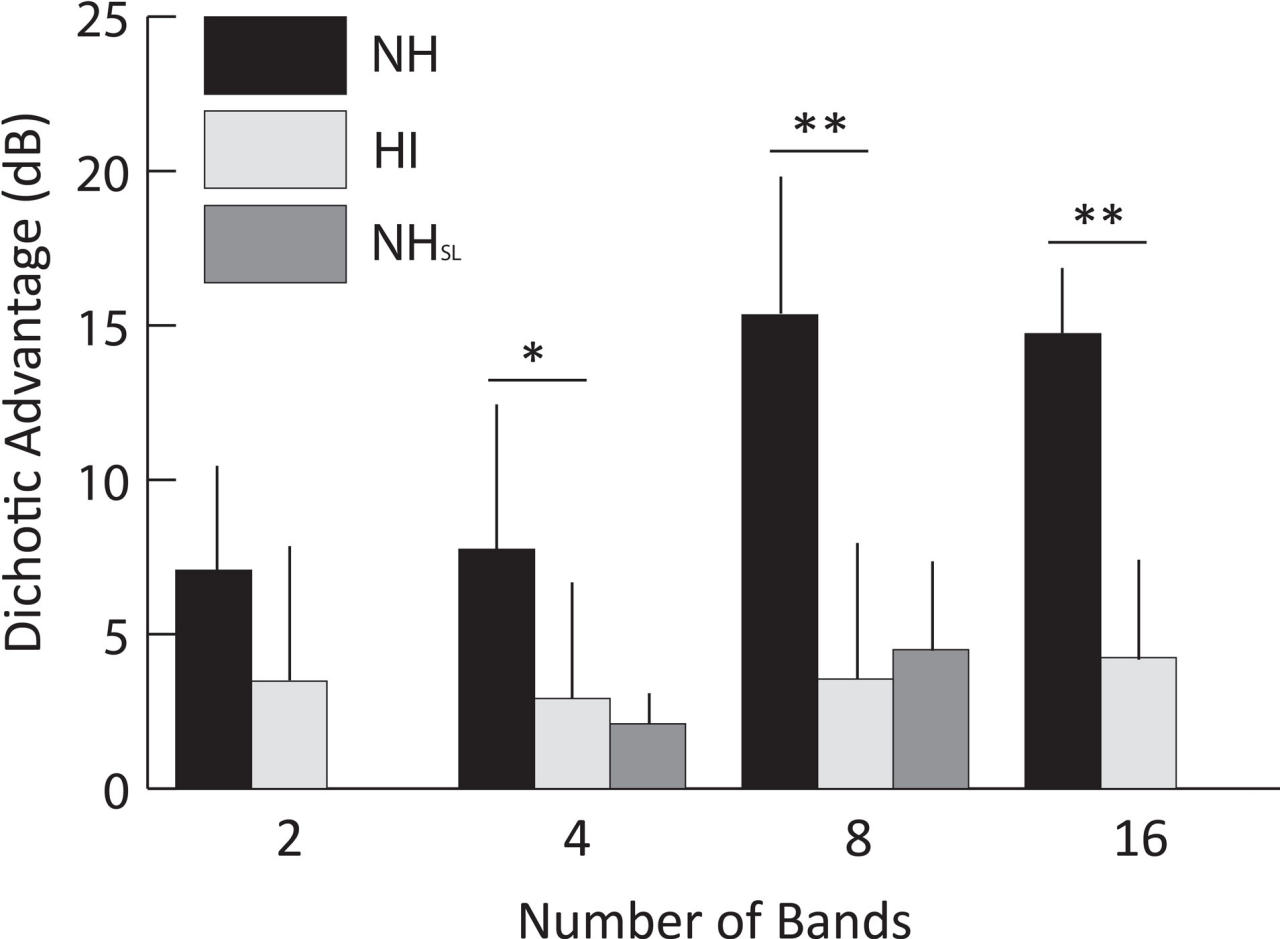
The effect of hearing loss on dichotic benefit. Dichotic benefit (i.e., the difference between Async-D and Async-M conditions) for hearing-impaired (HI) and normal hearing controls (NH and NH_SL_). Error bars indicate one standard deviation. Whereas the dichotic advantage increased significantly for NH listeners as band number increased, HI and NH_SL_ listeners received similar benefit at all band numbers (NH_SL_ group was only tested at 4 and 8 bands). Significant differences between groups are indicated by an asterisk (p < 0.05) or two asterisks (p < 0.001).

The differences in masking release between Async-D and Async-M conditions–referred to as dichotic advantage–are presented in Fig 5 for NH (black bars) and HI (shaded bars) listeners. The dichotic advantage was between 7.1 and 15.3 dB for the NH group, and between 2.9 and 4.2 dB for the HI group. A two-way ANOVA with two levels of group and four levels of number of bands resulted in a main effect of group (F_1,14_ = 23.5, p < 0.001), a main effect of number of bands (F_3,42_ = 12.4, p < 0.001), and a significant interaction (F_3,42_ = 8.8, p < 0.001). Simple main effects indicate that NH listeners had greater dichotic advantage than those with hearing loss for 4 bands (p < 0.05), 8 bands (p < 0.001) and 16 bands (p < 0.001), but not for 2 bands (p = 0.096). This is due to the fact that the dichotic advantage increased with number of bands for the NH group, but did not increase as much (if at all) for the HI group. Again, it is possible that the magnitudes of differences across conditions in the HI listeners are limited due to their smaller maximum masking release in the synchronous modulation condition. This was possible to assess from the NH_SL_ group data, in which masking release in the Sync-M condition was more comparable between the listener groups. Dichotic advantage was analyzed for the NH_SL_ and HI groups by submitting data to a two-way ANOVA with factors of group and number of bands. Although there was a significant main effect of number of bands (F_1, 15_ = 5.0, p < 0.05), there was no significant effect of group or interaction between group and number of bands. The lack of an interaction is particularly interesting in light of the fact that HI listeners performed more poorly than the NH_SL_ group in the 4-band, Async-M condition. This reveals that although HI listeners had more difficulty glimpsing speech in the presence of neighboring noise bands, the degree of benefit they received from dichotic listening was comparable to the NH listeners tested at a comparable sensation levels.

### Evaluation of spectro-temporal integration

Control measures taken in the study were useful in assessing the possibility that a listener was attending just to a subset of bands–either the even or the odd bands–in the Async conditions, thereby not actually integrating across frequency *and* time. Performance in the Async-D conditions was uniformly better than either Async-D-ODD or Async-D-EVEN control conditions for both groups. The difference in SRTs between the Async-D condition and the better of the two control conditions ranged from 5.9 to 10.2 dB for NH subjects and from 3.5 to 6.3 dB for HI subjects, depending on the number of bands. This result supports the interpretation that speech perception in the Async-D condition was not based on cues present in either subset of bands presented to a single ear, but rather relied on cues distributed across ears.

Recall that in the Async-D-EVEN and Async-D-ODD conditions, the noise-only ear received bands of noise that were modulated out-of-phase relative to the masker modulation in the ear presented with the speech signal. We compared the Control-D and Control-M measures at 8 bands to assess the effect of including asynchronously modulated masker bands contralateral to the speech-plus-noise stimulus. Masking release in the monaural control conditions was 3.5 dB greater than in the dichotic control conditions for NH listeners; this difference was 4.0 dB for HI listeners. From these results, it appears that having a modulated masker in non-overlapping frequency regions in one ear can mask speech in the other ear. The across-ear masking indicated by the comparison between the monaural and dichotic control conditions may help explain why listeners do not obtain as much release from masking in the Async-D conditions as they do in the Sync condition. That is, although the effects of masking spread have been reduced in the Async-D condition, there appears to be some other factor limiting performance in the dichotic condition. One possibility is that a contralateral masker affects perceptual weighting based upon masker modulation phase and listening in the dips [41]. Because the masker modulation phase in the contralateral ear was antiphasic with respect to the modulation in the speech ear, it is possible that the contralateral masker resulted in “miscuing” that blunted the benefit of improved SNR associated with masker dips in the speech ear.

## Discussion

### Factors contributing to reduced masking release in HI listeners

#### Effect of audibility

Overall, HI listeners in the present study had less masking release than the NH group. Data from the NH_SL_ group provided some indication that much of the difference between HI and NH listeners could be attributed to audibility. Previous studies have shown that HI listeners benefit less from masker amplitude modulation than NH listeners when stimuli are presented at equal levels [17, 42–45], especially for single syllable stimuli [46]. Although some of these results can be explained by reduced audibility in the masker dips for low-level speech cues, it has been suggested that other contributors are poor temporal resolution [10, 46] or poor frequency resolution [14, 17]. In the equivalent-intensity configuration, overall levels were fixed at 85 dB SPL; however, speech signals could fall well below that in some conditions, and low-level speech cues can be very important for identification [47]. For example, the target speech is approximately 60 dB SPL at -25 dB SNR, the approximate best SRT obtained in NH listeners; while a 60-dB-SPL target would be detectable in quiet for all HI listeners tested, some of the low-level cues would likely be inaudible, limiting those listeners’ ability to glimpse speech in the modulated maskers.

Another factor to consider when comparing masking release between listening groups is the threshold difference in the reference condition. Generally, NH listeners achieve larger masking release when the baseline SNR is more negative [48], which is related to the performance intensity function of speech perception in noise [7]. The performance-intensity function indicates how much change in speech recognition is associated with a change in level–at medium levels, small changes in level will lead to large performance differences, whereas at low and high levels, small changes in level do not affect performance as much. Whereas numerous studies have shown that HI listeners are less able to benefit from the introduction of masker fluctuation compared to NH listeners [10, 17, 42], Bernstein and Grant [7] note that these particular studies were undermined by a confound between group differences in the baseline SNR. In the present study, baseline SNRs were not found to be significantly different between NH and HI groups, so this issue was less of a concern. The lack of baseline SRT differences between the NH and HI listeners is somewhat surprising because most previous studies have found elevated masked SRTs in listeners with sensorineural hearing loss [7, 49, 50]. For elevated presentation levels such as ours, however, NH listeners have been shown to be more closely aligned with HI listeners in masked-speech performance [47]. This possibility is supported by the data from the NH_SL_ group which did differ significantly from the HI group in baseline SRT (-2.2 dB SNR and 0.3 dB SNR, respectively). However, the two groups did not consistently differ in their benefit from masker modulation, so this is a clear divergence from previous reports. Nevertheless, we considered two additional factors that may have contributed to the lack of a baseline SRT difference between HI and NH groups. First, steeply sloping hearing loss has a greater detrimental effect on consonant recognition than gradually sloping or flat losses [51, 52]. The HI listeners in the present study had relatively flat audiograms, which would be associated with relatively modest effects of hearing loss on masked SRTs. Second, the use of pink noise in the present study could have affected performance. Whereas speech-spectrum noise falls off at approximately 8 dB/octave [53], pink noise falls off at 3 dB/octave. Because pink noise is relatively less effective at masking low- than high-frequency speech features, this masker could increase listeners’ reliance on low-frequency cues. For the HI listeners in the present study, the hearing loss was relatively mild at low frequencies, which could have played a role in their good performance relative to NH listeners. This possibility is undermined, however, by the finding that flat mild/moderate hearing loss reduces performance for word recognition in pink noise [50].

#### Effect of frequency selectivity

Performance by all listeners was better for the asynchronous modulation conditions when stimuli were presented dichotically rather than monaurally. On average, this dichotic advantage was 7–15 dB for the NH listeners, which was even larger than previously seen (roughly 5–8 dB benefit in [24]). One methodological difference between studies that might account for this difference is presentation level. The current procedure presented stimuli at an overall level of 85 dB SPL (before modulation or separation of bands), whereas the previous experiment fixed the target level at 55 dB SPL and varied the masker level to estimate threshold. Therefore it should not be as surprising, considering that masking release has been shown previously to be smaller at lower intensities for both the synchronously- [10] and asynchronously-modulated maskers (e.g., Experiment 2 in [24]). Consequently, there was no difference between HI and NH_SL_ groups in dichotic advantage. At similar sensation levels, HI listeners were able to integrate across spectro-temporal glimpses as well as normal-hearing controls when negative effects of peripheral spread of masking were removed. The lone difference between HI and NH_SL_ groups was in the 4-band, monaural Async condition. Whereas each group performed equally poorly in the 8-band, monaural Async condition (i.e., no better than the 3.2 dB level difference accounted for by the modulation), the HI listeners also performed poorly in the 4-band case. This result was another clear indication that for these spectrally wide glimpsing regions, audibility alone could not account for the extent of the poor performance by the HI group. Indeed, the poor performance of the HI listeners in the 4-band monaural Async condition was probably due to reduced frequency selectivity. This interpretation is consistent with the finding that performance improved when the stimuli were presented dichotically.

### Possible clinical applications of dichotic listening

The results of this study have important implications for hearing aid design. In quiet settings, most aided HI listeners with mild-to-moderate sensorineural hearing loss have minor difficulty following a conversation. However, the same listeners often complain that it is difficult to follow speech in noisy environments. Traditional hearing aids with advanced noise-reduction processing [54] have been largely ineffective in improving speech understanding in noise. One of the obvious factors contributing to this phenomenon is that amplification has the negative effect of adding gain to all incoming sounds, including the unwanted noise. Therefore, supplementary strategies, like dichotic presentation, could be utilized to limit the influence unwanted noise has on speech perception.

There have been previous attempts to use dichotic presentation to improve speech identification in hearing-aid or cochlear implants users [25–28, 55], and the current study provides additional support for this approach. However, there could be unintended consequences of removing crucial binaural spatial cues, such as interaural time or level differences [29], so further study in spatially diverse settings is still needed. Along with the support of amplification, dichotic presentation should be considered as an appropriate strategy for improving speech-in-noise performance.

## Conclusions

Previous studies using monaural Async maskers [2, 24] showed a decrease in the ability to benefit from masker modulation with increasing number of bands. One possible reason for this was increased spread of masking effects as the band number increased. Ozmeral *et al*. [24] aimed to reduce the possible deleterious effects of spread of masking by presenting neighboring spectral bands to separate ears [25]. The result was 5-to-8-dB better SRTs across all band conditions in the Async-D condition relative to the Async-M condition. The current study replicated NH listener data reported by Ozmeral *et al*. [24], and added the HI group to determine whether listeners with sensorineural hearing loss could also benefit from dichotic listening in the presence of an Async masker. Because HI listeners tend to have poorer-than-normal frequency selectivity [13–17], it was hypothesized that masking release would be greatly reduced or absent in a monaural asynchronous masker, but that dichotic presentation could facilitate masking release.

At equal presentation levels, listeners with sensorineural hearing loss had less masking release than age-matched normal-hearing listeners for speech presented in synchronously- and asynchronously-modulated noise. Testing at similar sensation levels between groups, however, indicated that masking release was closely associated with overall audibility. Importantly, the dichotic listening benefit was equivalent between HI and NH_SL_ groups. These results are consistent with an interpretation that the reduced masking release shown by the HI listeners in monaural asynchronously-modulated noise is due to a combination of reduced audibility and poor frequency selectivity, and that amplification along with dichotic stimulation may provide the best outcomes for speech in spectro-temporally complex noise.
